# A prospective observational study of all-cause mortality in relation to serum 25-OH vitamin D_3_ and parathyroid hormone levels in patients with type 2 diabetes

**DOI:** 10.1186/s13098-015-0049-9

**Published:** 2015-06-12

**Authors:** Pär Jennersjö, Hans Guldbrand, Stefan Björne, Toste Länne, Mats Fredrikson, Torbjörn Lindström, Magnus Wijkman, Carl Johan Östgren, Fredrik H. Nystrom

**Affiliations:** Department of Medical and Health Sciences, Linköping University, SE 581 83 Linköping, Sweden; Department of Clinical and Experimental Medicine, Faculty of Health Sciences, Linköping University, Linköping, Sweden; Department of Internal Medicine and Department of Medical and Health Sciences, Linköping University, Norrköping, Sweden

**Keywords:** Arteriosclerosis, Calcium, Mortality, Parathyroid hormone, Type 2 diabetes, Vitamin D

## Abstract

**Background:**

Low levels of vitamin D have been related to increased mortality and morbidity in several non-diabetic studies. We aimed to prospectively study relationships between serum 25-OH vitamin D_3_ (vitamin D) and of serum parathyroid hormone (PTH) to total mortality in type 2 diabetes. We also aimed to compare the levels of these potential risk-factors in patients with and without diabetes.

**Methods:**

The main study design was prospective and observational. We used baseline data from 472 men and 245 women who participated in the “Cardiovascular Risk factors in Patients with Diabetes—a Prospective study in Primary care” study. Patients were 55–66 years old at recruitment, and an age-matched non-diabetic sample of 129 individuals constituted controls for the baseline data. Carotid-femoral pulse-wave velocity (PWV) was measured with applanation-tonometry and carotid intima-media thickness (IMT) with ultrasound. Patients with diabetes were followed for all-cause mortality using the national Swedish Cause of Death Registry.

**Results:**

Levels of vitamin D were lower in patients with diabetes than in controls, also after correction for age and obesity, while PTH levels did not differ. Nine women and 24 men died during 6 years of median follow up of the final cohort (*n* = 698). Vitamin D levels were negatively related to all-cause mortality in men independently of age, PTH, HbA1c, waist circumference, 24-h systolic ambulatory-blood pressure (ABP) and serum-apoB (*p* = 0.049). This finding was also statistically significant when PWV and IMT were added to the analyses (*p* = 0.028) and was not affected statistically when medications were also included in the regression-analysis (*p* = 0.01). In the women with type 2 diabetes, levels of PTH were positively related with all-cause mortality in the corresponding calculations (*p* = 0.016 without PWV and IMT, *p* = 0.006 with PWV and IMT, *p* = 0.045 when also adding medications to the analysis), while levels of vitamin D was without statistical significance (*p* >0.9).

**Conclusions:**

Serum vitamin D in men and serum PTH in women give prognostic information in terms of total-mortality that are independent of regular risk factors in addition to levels of ABP, IMT and PWV.

**Trial registration:**

ClinicalTrials.gov: NCT01049737

## Introduction

Vitamin D is stored in adipose tissue and liver in an inactive form, and serum levels of vitamin D are negatively related to adiposity [1]. Prospective observational studies have shown inverse associations between serum vitamin D and future risk of hyperglycemia, insulin resistance and incident diabetes [2–5]. Furthermore, low levels of serum vitamin D (25-hydroxyvitamin D_3_) have been linked with an increased risk for mortality in different non-diabetic populations [6, 7]. In experimental studies vitamin D has been shown to reduce activity in the renin angiotensin system [8] and inflammation has also been inhibited by action of vitamin D [9, 10] suggesting causality between low levels of this vitamin and increased prevalence of cardiovascular disease. However, arguing against a simple inverse relationship between levels of vitamin D and risk for disease was the quite recent finding of increased mortality rates in two large studies among subjects with particularly high levels of vitamin D [11, 12]. Deficiency of vitamin D leads to increased levels of parathyroid hormone levels (PTH) which allows release of calcium that is stored in bone tissue. High levels of PTH have been linked with increased arterial calcification in a population-based study in Sweden [13] and it is well known that secondary hyperparathyroidism is an indicator of poor outcome in renal diseases in which activation of vitamin D is deficient [14].

New and clinically useful markers of risk are of essence in high risk states such as type 2 diabetes. Recently it was shown that patients with type 2 diabetes and low vitamin D levels had an increased risk for all-cause mortality in a study with a median follow-up time of 15 years [15], and similar results have been obtained in patients with type 1 diabetes [16]. However, information on levels of PTH, for determination of potential primary or secondary hyperparathyroidism, was lacking in these trials and no relationship between vitamin D levels and degree of obesity was found [15, 16].

The major cause of death in type 2 diabetes is cardiovascular disease. One of the strongest predictors of such manifestations of arteriosclerosis is the aortic pulse wave velocity (PWV), which is a non-invasive measure of the degree of arterial calcification [17]. Yet another non-invasive measure of degree of vascular disease is the thickness of the intima and media (IMT) of carotid arteries in patients, which can be readily measured by ultrasound [18].

We herein report a study of 698 patients with type 2 diabetes with a median follow-up of six years in which serum levels of vitamin D, calcium and PTH were analyzed. We also studied interaction with arterial calcification at baseline by measurement of carotid-femoral PWV and carotid IMT. Our main aim was to assess if levels of baseline serum vitamin D would give independent information about total mortality independently of common clinical risk-markers. Furthermore we wanted to study if such potential associations of levels of vitamin D with total mortality would be affected by addition of PWV and IMT in this cohort of patients with high risk for cardiovascular disease.

## Research design and methods

### Patients and non-diabetic controls

We analyzed baseline data from 717 patients, who participated in a community-based cohort, Cardiovascular Risk factors in Patients with Diabetes—a Prospective study in Primary care, acronym CARDIPP. This prospective observational study was launched in November 2005 and recruitment of the participants was completed in 2008. All these patients had type 2 diabetes and were between 55–66 years old when they were consecutively recruited from 22 different primary health care centers in the counties of Östergötland and Jönköping, Sweden. Detailed information about the structure and results from CARDIPP has been described previously [19, 20]. The centers were located in different demographic areas and differed in size. However, the model of treatment and care of diabetes was organized similarly and the primary care centers adhered to the same national guidelines of diabetes care. Age-matched participants from the CAREFUL (CArdiovascular REFerence popULation) study served as non-diabetic controls. As described previously [21], study participants in CAREFUL had been randomly selected from a population registry of individuals aged 50–70 years who resided within the catchment area of Linköping University Hospital. Individuals with previously known or newly discovered diabetes mellitus (defined as either a self-reported history of diabetes mellitus, current use of anti-diabetic medications or a fasting plasma glucose ≥ 7.0 mmol/l at base-line) were not eligible for participation in the CAREFUL study, and neither were individuals with a family history or known diagnosis of aortic aneurysm. The participants in CAREFUL were not followed prospectively, but underwent similar base-line examinations as the patients who participated in CARDIPP. Recruitment to the CAREFUL study took place between February 2008 and March 2010. Among the 185 CAREFUL participants, controls for this study consisted of all participants aged 54–66 years for which data concerning vitamin D had been obtained. This yielded a control sample of 129 non-diabetic individuals.

### Anthropometric measurements

Nurses especially dedicated to treatment of diabetes at the primary health care centers or at the Endocrine Clinic at Linköping University Hospital measured height to the nearest cm and weight to the nearest 0.1 kg with the patients wearing light indoor clothing. Waist circumference (WC) was measured with the patient standing, after a regular expiration, to the nearest cm, midway between the lowest rib and the iliac crest. A standardized medical history was taken that included medication, vitamin- and other supplements and smoking habits.

### Laboratory analyses

Blood was drawn in the morning after a 10 h over-night fast. Plasma glucose was analyzed according to routines at the primary health care centers. Other routine tests, lipids and HbA1c etc., were analyzed at the Department of Clinical Chemistry at Linköping University Hospital. Levels of cholesterol, HDL and triglycerides were measured with enzymatic methodology and spectrophotometry, Selectra E, Vital Scientific, Netherlands/Triolab. Levels of LDL cholesterol were calculated by Friedewalds formula: LDL cholesterol = total cholesterol − HDL cholesterol − 0.45 × fS – triglycerides. HbA1c was analyzed according to the Swedish Mono-S HPLC, all HbA1c values were converted to DCCT standard values using the formula: HbA1c DCCT = 0.923 × HbA1c (Mono − S) + 1.345 (R2 = 0.998) and reported in IFCC units. Levels of apoB and apoA-I were measured by immunoturbidimetric assays, Advia 1800, DakoCytomation, Glostrup, Denmark. CRP was also measured by immunoturbidimetric assays, Advia 1800, Siemens Diagnostic Medical Solutions, Erlangen, Germany, with a detection level of 0.12 mg/L. PTH and 25-OH-vitamin D_3_ were analyzed using chemoluminiscense on a Cobas® e602 unit (Roche Diagnostics Scandinavia AB, Bromma, Sweden). Serum levels of calcium were measured on a KonelabTM PRIME 30i equipment (Thermo Fischer Scientific Inc, Waltham, MA, USA). Albumin-corrected calcium was calculated as total calcium + 0.02 * (40 - albumin).

Coefficients of variation (CV) were calculated for the analyses performed in the CARDIPP cohort. For 25-OH-vitamin D_3_, the CV was 8.7 % at a mean level of 41 nmol/L and 3.5 % at a mean level of 91 nmol/L, and the CVs for PTH were 5.8 % at a mean level of 3.6 pmol/L and 4.6 % at a mean level of 13.1 pmol/L, respectively. At a mean calcium level of 0.78 mmol/L the CV was 4.6 % and at a mean level of calcium of 1.50 mmol/L the CV was 3.1 %. For apoB and apoA-I, CVs were 1.2 % and 1.8 %, respectively and for CRP the CV was 1.6 %.

### Clinical physiological investigations and pedometry

Determination of PWV was done at the Department of Physiology, Linköping University Hospital, Linköping and at the County Hospital Ryhov, Jönköping, as described previously [19, 20]. In brief, the aortic PWV was measured with applanation tonometry (SphygmoCor® system, model MM3, AtCor Medical, Sydney, Australia) over the carotid and femoral arteries. The aortic pulse wave transit times were measured by electrocardiogram-guided readings of the femoral arterial pulse waves, using the carotid arterial pulse wave as the reference site. The surface distances were measured from the suprasternal notch to the carotid and femoral measurement sites, respectively. PWV was calculated by dividing the surface distance with the pulse wave transit time yielding m/s.

IMT of the carotid arteries was evaluated using B-mode ultrasound. A digital ultrasound system (ATL HDI 5000, Bothell, WA, USA) equipped with a broadband linear transducer (L12-5) was used for scanning the carotid artery in the longitudinal section. A 10 mm long section of the common carotid artery in the proximity of the carotid bulb was selected to obtain mean lumen diameter and far wall IMT. Three consecutive frozen images with focus on lumen-intima echo and media-adventitia echo of the far arterial wall were used. The digital B-mode images were subsequently transferred to a computer with dedicated software for off-line measurement of IMT (Artery Measurement System II, Image and Data Analysis, Gothenburg, Sweden). Calibration and subsequent measurement was performed by manually at both study locations (Jönköping and Linköping) tracing a cursor along the leading edge of the intima-lumen echo of the near wall, leading edge of the lumen- intima echo and media-adventitia echo of the far wall. During analysis, the measurement window was hidden for the reader and values were saved in a text file. Mean values of IMT from both the right and the left sides were used in all analyses.

Ambulatory BP measurement devices (Spacelab 90217, Spacelabs Inc., Redmond, Washington, USA) were set to measure the BP at 20-min intervals for 24 h as reported earlier in detail [20]. Physical activity was measured in a subset of the cohort with diabetes (*n* = 343) with waist-mounted pedometers during three consecutive days and the number of steps/day were calculated as reported earlier [19].

### All-cause mortality in patients with diabetes

The participants with diabetes were followed for all-cause mortality until December 31, 2012, by linkage with the Swedish Cause of Death Registry (The National Board of Health and Welfare, Stockholm, Sweden) using their unique personal identification numbers.

### Ethics

All participants gave written informed consent prior to participating in the study. The study, which complied with the declaration of Helsinki, was approved by the Regional Ethical Review Board in Linköping, Sweden.

### Statistics

IBM SPSS statistics 21and 22 (IBM corporation, Somers, NY, USA) were used for statistics. In the survival analyses, Cox regression proportional hazard model was used. The statistical significance of between-group differences were tested with unpaired t-tests for continuous variables, and with the Chi-Square test for dichotomous variables. Estimates of adjusted between-group differences were obtained by multiple regression analysis. To assess strengths of correlations between continuous variables, Pearson correlation coefficients were calculated. Statistical significance was defined as *p* < 0.05.

## Results

### Vitamin D and PTH levels according to diabetes status

Compared with 129 non-diabetic controls (67 women and 62 men), the 717 patients with type 2 diabetes had significantly lower levels of vitamin D, but there was no significant difference concerning levels of PTH (Table 1). Expectedly, patients with diabetes also had significantly higher BMI, waist circumference and ambulatory systolic blood pressure, and significantly higher PWV and IMT (Table 1). Mean HbA1c (MonoS) in the patients was 6.08 ± 1.1 % (52.9 ± 12 mmol/mol). In a multivariate regression model, the association between prevalent diabetes and lower vitamin D levels remained significant after adjustment for age, gender and BMI (estimated adjusted between-group difference:15.1 nmol/l, 95 % CI 10.0 – 20.2). Substitution of BMI for waist circumference did not alter the significance of the association between prevalent diabetes and lower vitamin D levels (estimated adjusted between-group difference: 15.3 nmol/l, 95 % CI 10.5 – 20.1 nmol/l). Women with diabetes had a mean PTH of 46.0 ± 16 ng/l and the corresponding level in men was 47.4 ± 18 ng/l (p = 0.30 for comparison between genders).

**Table 1:** Baseline characteristics of patients with type 2 diabetes, and age-matched non-diabetic controls

Variable	Patients (*n* = 717)	Controls (*n* = 129)	*p*
Age (years)	60.7 ± 3.1	59.6 ± 4.4	<0.01
Female gender, n (%)	245 (34 %)	67 (52 %)	<0.01
BMI (kg/m^2^)	30.2 ± 4.7	25.8 ± 3.1	<0.01
Waist circumference (cm)	104.5 ± 11.9	92.7 ± 11.4	<0.01
Sagittal abdominal diameter (cm)	25.6 ± 3.8	22.4 ± 3.6	<0.01
Total cholesterol (mmol/l)	4.7 ± 1.0	5.8 ± 1.0	<0.01
HDL cholesterol (mmol/l)	1.3 ± 0.3	1.6 ± 0.4	<0.01
LDL cholesterol (mmol/l)	2.7 ± 0.8	3.5 ± 0.9	<0.01
Triglycerides (mmol/l)	1.8 ± 1.1	1.3 ± 0.6	<0.01
Office systolic blood pressure (mmHg)	137.2 ± 16.4	132.4 ± 15.6	<0.01
Office diastolic blood pressure (mmHg)	79.9 ± 10.0	84.3 ± 9.1	<0.01
Ambulatory systolic blood pressure (mmHg)	129.8 ± 13.7	123.4 ± 13.9	<0.01
Ambulatory diastolic blood pressure (mmHg)	76.4 ± 8.1	76.3 ± 8.8	NS
25-OH vitamin D (nmol/l)	50.9 ± 22.0	68.3 ± 26.3	<0.01
PTH (ng/l)	47.1 ± 17.4	46.2 ± 14.4	NS
Albumin-corrected serum calcium (mmol/l)	2.25 ± 0.17	2.30 ± 0.08	<0.01
PWV (m/s)	10.3 ± 2.1	9.2 ± 2.3	<0.01
IMT (mm)	0.74 ± 0.2	0.68 ± 0.2	<0.01
Statin (%)	55	12	<0.01
Acetylsalicylic acid (%)	29	9	<0.01
Thiazide diuretic (%)	10	8	0.47
ACE inhibitor or ARB (%)	43	13	<0.01
Calcium antagonist (%)	15	6	<0.01
Beta blocker (%)	35	15	<0.01
Insulin (%)	32	-	-
Metformin (%)	53	-	-
Rosiglitazone or pioglitazone (%)	5	-	-
Sulfonylurea or glinide (%)	15	-	-
Acarbose (%)	2	-	-

Data are presented as means ± SD or as actual number (n). P values correspond to the differences between the two groups by unpaired t-tests for continuous variables and Chi-square test for medications. Since the controls per definition did not use glucose lowering medications, no p-values were calculated between groups for these variables

Number of participants with missing data: 32 (BMI), 21 (waist circumference), 28 (sagittal abdominal diameter), 40 (total-cholesterol), 43 (HDL-cholesterol), 72 (LDL-cholesterol), 46 (triglycerides), 32 (office blood pressures), 69 (ambulatory blood pressures), 20 (PTH), 4 (corrected serum calcium), 64 (PWV), 24 (IMT)

### Baseline correlates of vitamin D levels in patients with diabetes

Nineteen subjects with diabetes reported taking vitamin supplements on a regular basis that was judged likely to contain vitamin D and these participants were excluded from the correlation and survival analyses, yielding a final cohort of 698 patients with type 2 diabetes. Among them, there were no cases of primary hyperparathyroidism, i.e. increased levels of both PTH and calcium, and no patient had estimated glomerular filtration rate < 30 ml/min/1.73 m^2^. There was a negative linear relationship between vitamin D levels and BMI (r = -0.146 p < 0.0001) and also with WC (r = -0.147, *p* < 0.0001) in the total diabetes cohort. PTH levels and calcium (corrected for albumin) were also related to vitamin D levels (PTH: r = -0.161, < 0.0001, calcium: r = 0.077 p = 0.042, respectively). Mean systolic 24-h ambulatory blood pressure correlated negatively with vitamin D (r = -0.132, p = 0.001, diastolic BP, *p* = 0.28) while levels of CRP did not (*p* = 0.47, after exclusion of values above 5 mg/l considered to be outliers). Vitamin D levels did not correlate significantly with PWV (*p* = 0.07) or IMT (p = 0.83). There were no statistically significant correlations between vitamin D levels and time since the diagnosis of diabetes (*p* > 0.46 in men and women) or habitual walking distances as determined by pedometry (p > 0.81 in men and women). Vitamin D levels were not elevated in seasons with high exposure to the sun (spring and summer) compared with periods of the year with less strong sunlight in Sweden, as analyzed in different ways (either as for example summer versus winter or when comparing all four majors seasons of the year). Vitamin D levels did not differ in patients treated with statins when compared with the rest of the cohort (*p* > 0.67 in both genders). Vitamin D levels correlated negatively with HbA1c in men and women (men: r = -0.10, p = 0.041, women: r = -0.14, *p* = 0.04). Women who had HbA1c below 52 mmol/mol had higher levels of vitamin D than those with higher levels of HbA1c (52.2 ± 22 nmol/l compared with 46.1 ± 19 nmol/l, p = 0.03) but this finding was not present in men (HbA1C < 52 mmol/mol: 52.4 ± 20 nmol/l, HbA1C ≥ 52 mmol/mol: 50.5 ± 25 nmol/l, *p* = 0.38). In men there was a weak negative correlation between glomerular filtration rate, calculated according to MDRD [22, 23], and vitamin D levels (men: r = -0.13, *p* = 0.006, women: r = -0.064, *p* = 0.34).

### Vitamin D levels, PTH levels and mortality in patients with diabetes

Cox regression was performed separately in men and women since gender differences are well established among several cardiovascular risk factors and so are indices of adiposity. During the follow-up period of a median of six years, 24 men and 9 women died. When entering major risk factors in Cox regression analyses, vitamin D levels were negatively related to all-cause mortality in men with diabetes, independently of baseline age, PTH, HbA1c, waist circumference, mean 24 h systolic ambulatory blood pressure and apoB levels (Table 2). This major finding of increased mortality related to low levels of vitamin D was also statistically significant when baseline carotid-femoral PWV and carotid IMT were added to the calculations (Table 3) and also when medications as in Table 1 were added to the Cox-regression (*p* = 0.01). Levels of PTH were not significantly associated with mortality in men with diabetes (Tables 2 and 3). In women with diabetes, on the other hand, levels of PTH were positively related to all-cause mortality when performing the corresponding analyses, while vitamin D levels were without such statistical significance (Tables 4 and 5). This increased risk related to PTH in women was statistically significant also when medications as in Table 1 were added to the Cox regression analysis based on data as in Table 5 (*p* = 0.045).

**Table 2:** Cox regression analyses of total mortality in men

Variable	Hazard ratio (CI for one unit)	*P* value
Age (years)	1.023 (0.895-1.117)	0.737
Waist circumference (cm)	1.008 (0.972-1.045)	0.667
HbA1c (mmol/mol)	0.997 (0.961-1.034)	0.858
Apo B (g/l)	0.490 (0.051-4.717)	0.537
Systolic mean ABP (mmHg)	0.975 (0.944-1.007)	0.126
PTH (ng/l)	1.003 (0.981-1.026)	0.780
25-OH vitamin D (nmol/l)	0.979 (0.958-1.000)	0.049

Regression analysis of total mortality in relation to levels of serum vitamin D adjusted for age, waist circumference, HbA1c, apoB1, mean systolic 24 h ambulatory blood pressure levels and serum PTH in men

**Table 3:** Cox regression analyses of total mortality in men including correction for PWV and IMT

Variable	Hazard ratio (CI for one unit)	*P* value
Age (years)	1.005 (0.872-1.159)	0.942
Waist circumference (cm)	0.989 (0.947-1.034)	0.626
HbA1c (mmol/mol)	0.996 (0.957-1.037)	0.852
Apo B (g/l)	0.597 (0.056-6.409)	0.670
Systolic mean ABP (mmHg)	0.970 (0.933-1.009)	0.129
IMT (mm)	5.417 (0.953-30.8)	0.057
PWV (m/s)	1.085 (0.855-1.377)	0.501
PTH (ng/l)	0.994 (0.956-1.022)	0.666
25-OH vitamin D (nmol/l)	0.973 (0.95-0.997)	0.028

Regression analysis of total mortality in relation to levels of serum vitamin D adjusted for age, waist circumference, HbA1c, apoB1, mean systolic 24 h ambulatory blood pressure levels, serum PTH and also with addition of carotid femoral PWV and carotid IMT

**Table 4:** Cox regression analyses of total mortality in women

Variable	Hazard ratio (CI for one unit)	*P* value
Age (years)	1.120 (0.878-1.429)	0.362
Waist circumference (cm)	0.994 (0.940-1.052)	0.846
HbA1c (mmol/mol)	1.023 (0.957-1.094)	0.499
Apo B (g/l)	0.618 (0.018-21.61)	0.790
Systolic mean ABP (mmHg)	1.005 (0.956-1.057)	0.833
PTH (ng/l)	1.037 (1.007-1.068)	0.016
25-OH vitamin D (nmol/l)	1.001 (0.968-1.036)	0.942

Regression analysis of total mortality in relation to levels of serum vitamin D adjusted for age, waist circumference, HbA1c, apoB1, mean systolic 24 h ambulatory blood pressure levels, and serum PTH

**Table 5:** Cox regression analyses of total mortality in women including correction for PWV and IMT

Variable	Hazard ratio (CI for one unit)	*P* value
Age (years)	1.184 (0.864-1.624)	0.294
Waist circumference (cm)	0.962 (0.893-1.036)	0.300
HbA1c (mmol/mol)	1.042 (0.976-1.114)	0.218
Apo B (g/l)	13.66 (0.250-745.5)	0.200
Systolic mean ABP (mmHg)	1.002 (0.941-1.066)	0.956
IMT (mm)	44.58 (0.305-6511.2)	0.135
PWV (m/s)	1.042 (0.694-1.564)	0.843
PTH (ng/l)	1.049 (1.014-1.085)	0.006
25-OH vitamin D (nmol/l)	1.000 (0.963-1.038)	0.990

Regression analysis of total mortality in relation to levels of serum vitamin D adjusted for age, waist circumference, HbA1c, apoB1, mean systolic 24 h ambulatory blood pressure levels, serum PTH and also with addition of carotid femoral PWV and carotid IMT

Cox regression based on four groups of vitamin D levels, < 25 nmol/l, 25-49 nmol/l, 50-75 nmol/l and >75 nmol/l, did not show statistical significance in terms of mortality prediction in women (*p* = 0.9) but it bordered on significance in men (*p* = 0.053) when performed with other data as in Tables 2 and 4, respectively. Seventeen women (7.2 %) and 38 men (8.2 %) had vitamin D < 25 nmol/l, i.e. the prevalence of vitamin D deficiency was similar in men and women (Chi-Square, *p* = 0.65).

**Fig. 1:**
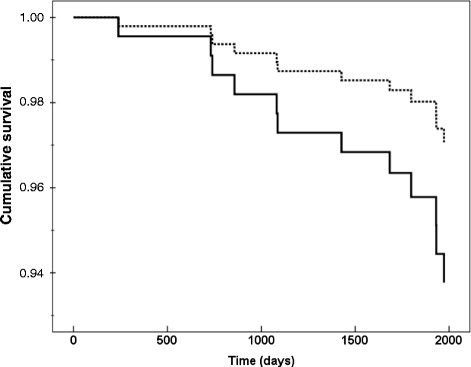
Cox regression analysis of total mortality in male patients with type 2 diabetes in relation to vitamin D tertiles. Data are shown for upper (dashed line) and lower tertiles (continuous line) of levels of vitamin D adjusted for parathyroid hormone levels, HbA1c, waist circumference, age, 24-h systolic ambulatory blood pressure, serum-apoB, carotid-femoral PWV and carotid IMT

**Fig. 2:**
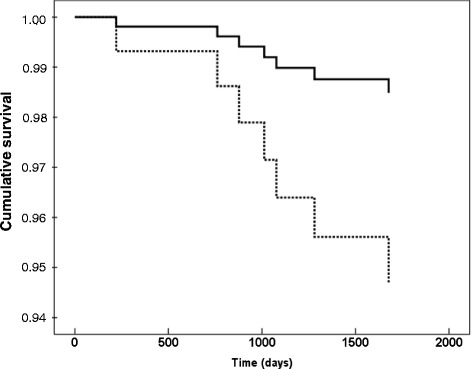
Cox regression analysis of total mortality in women with type 2 diabetes in relation to levels of parathyroid hormone levels. The dashed line represents upper tertile and the continuous line the risk in patients with levels in the lower tertile of parathyroid hormone levels after adjustments for levels of vitamin D, HbA1c, waist circumference, age, 24-h systolic ambulatory blood pressure, serum-apoB, carotid-femoral PWV and carotid IMT

Figure 1 shows Cox regression analysis of total mortality in patients with type 2 diabetes in the upper and lower tertiles of levels of vitamin D in men, after adjustment for the risk factors as in Table 3. Figure 2 shows the corresponding analysis of mortality in women with type 2 diabetes, for levels of PTH in the higher and lower tertiles, after adjustments for the risk factors as in Table 5.

## Discussion

In contrast to earlier reports on relationships between vitamin D and mortality in patients with type 2 diabetes [15, 16], our analysis also incorporated data on corrected calcium and of serum PTH levels. It is of importance to have knowledge also about levels of PTH and calcium in conjunction with vitamin D to properly determine presence of primary or secondary hyperparathyroidism [24] and also since high PTH levels have been found to be an indicator of risk *per se* in renal disease and in the general non-diabetic population [13, 14]. Our new analyses confirmed an independent relationship between low vitamin D levels and all-cause mortality in men with diabetes. This relationship was still statistically significant also when two other well-established risk markers for mortality, PWV and carotid IMT, were added to the analyses. This suggests that vitamin D can be used as a surrogate marker of risk for mortality in male patients with type 2 diabetes. It does not, however, prove that vitamin D substitution reduces the risk in the studied cohort. Results from experimental studies have suggested causality between treatment with vitamin D by for example reduction of inflammatory activity and inhibition of the renin angiotensin system [8, 9], but we found no relationships between levels of vitamin D and CRP. However vitamin D levels did show an inverse relationship with systolic ABP levels which is in line with a lack of suppression of renin angiotensin system by vitamin D. Recent studies of effects of treatment with vitamin D in non-diabetic patients on blood pressure and vascular function have shown benefits in some [25, 26], but not all [27, 28] studies. A recent large meta-analysis did not find reduced cardiovascular disease or mortality by administration of vitamin D [29]. However, the incorporated studies were not specifically aimed for evaluation of mortality, nor were they dedicated to patients with type 2 diabetes.

In women with diabetes, we found no association between vitamin D levels and mortality. This raises the possibility that the impact of vitamin D levels on non-skeletal health is gender-dependent, an issue worthy of further investigations. It should be noted that increased incidence of cardiovascular disease has earlier been shown in non-diabetic Danish women who had vitamin D deficiency [30]. Our analyses revealed, on the other hand, that levels of PTH in women with diabetes were positively related with all-cause mortality independently of vitamin D, PWV and carotid IMT. This was not a consequence of primary hyperparathyroidism or renal failure since no such cases were diagnosed in the cohort. However, our data cannot discern whether this relationship was based on mildly elevated PTH levels due to relative lack of active vitamin D, but it does suggest that measurement of serum PTH can add information in the clinical evaluation of risk for mortality in women with type 2 diabetes.

Our finding that prevalent diabetes is associated with decreased vitamin D levels is consistent with previous findings from a large American epidemiological study [31]. Despite this, we found no significant impact on PTH levels by diabetes status in our study. The lower levels of vitamin D that were observed among the patients with diabetes in our study were not likely to be attributable to the increased measures of adiposity, since this finding remained significant after adjustment for either BMI or waist circumference. Our finding that vitamin D levels were not associated with PWV in patients with diabetes is in contrast to a previous report [32]. However, the previously reported associations focused on patients with type 2 diabetes and established vitamin D deficiency, whereas we included patients with a wide range of vitamin D levels. In men we found a weak, but statistically significant negative correlation between glomerular filtration rate and vitamin D levels. This would argue against that the two proteins FGF23 and megalin, which are involved in vitamin D metabolism [33–35], were affected by diabetic nephropathy and hence linked with the increased risk for mortality in our study. However, we did not specifically assess levels or activity of these proteins which could indeed be of interest in future studies of type 2 diabetes and vitamin D in humans.

To the best of our knowledge, this is the largest study to demonstrate a relationship between vitamin D levels and mortality in men with type 2 diabetes. Strengths in our study include registry data on mortality with no patients lost to follow-up. It is the first study of its kind that includes measures of PTH and corrected calcium in conjunction with vitamin D. We could also analyze data with respect to novel markers of atherosclerosis and vascular disease, such as PWV and carotid IMT. Weaknesses include a rather short follow-up time period and consequent low rates of mortality. However the short follow-up period suggests clinical relevance insofar that patients were indeed being cared for in a manner that is in accordance with current guidelines. This was confirmed by frequent treatment with statins and antihypertensive medication while mean levels of HbA1c and ambulatory blood pressures were generally acceptable. Also we were not able to analyze data with regard to significant renal failure, a condition that affects vitamin D metabolism, since no patients had estimated glomerular filtration rate < 30 ml/min/1.73 m^2^. We also acknowledge that data on estrogen replacement therapy was lacking. However, we assumed that patients were treated according to guidelines in this respect and thus that post-menopausal estrogen replacement therapy was only used for very short periods of time (months) in selected patients.

Although statin treatment has been associated with an increase in vitamin D levels [36], we found no such interactions between use of statins and high vitamin D levels in patients with diabetes. The lack of relationship between season of the year and vitamin D levels was most likely due to the fact that very few patients were investigated during summer in Sweden since the study was performed mainly at Linköping University where vacations among staff precluded much recruitment of patients in the study from May to September. The same conditions were present during a dark time of the year, since no investigations were done at the time of Christmas and the New Year.

In summary, we found that low vitamin D levels were associated with a significantly increased risk for premature mortality in men with type 2 diabetes. Likewise, high levels of PTH were associated with a significantly increased risk for premature mortality in women with type 2 diabetes. These associations were independent of both traditional and new markers of risk, such as ambulatory blood pressure, PWV and carotid IMT. Our observational data in patients with type 2 diabetes show that the finding of low vitamin D levels in men or of high PTH levels in women gives independent prognostic information of an increased risk for total mortality.

## Abbreviations

ABP: 24-h ambulatory blood pressure
ACE: Angiotensin converting enzyme
Apo B: Apolipoprotein B1 levels
ARB: Angiotensin II receptor antagonist
BMI: Body mass index
HDL: High density lipoprotein
IMT: Intima-media thickness of carotid arteries
LDL: Low density lipoprotein
PTH: Parathyroid hormone levels
PWV: Carotid-femoral pulse wave velocity
